# Geographical influences on the iodine status in pregnant women, neonates, and school-age children in China

**DOI:** 10.1186/s12937-020-0525-4

**Published:** 2020-01-21

**Authors:** Xiaoming Lou, Xiaofeng Wang, Guangming Mao, Wenming Zhu, Zhe Mo, Yuanyang Wang, Zhifang Wang

**Affiliations:** Division of Endemic Diseases, Department of Environmental Health, Zhejiang Provincial Center for Disease Control and Prevention, 3399 Binsheng Road, Binjiang District, Hangzhou city, 310051 Zhejiang Province China

**Keywords:** Iodine status, Thyroid-stimulating hormone, Pregnancy, Neonates, School-age children

## Abstract

**Background:**

Pregnant women, neonates, and school-age children are vulnerable to iodine deficiency. The iodine contents in the environment (drinking water and household salt for cooking) vary by geographical location in China. The aim of this study was to assess the iodine status in vulnerable groups from different geographical zones and analyze the iodine content in household salt and drinking water from these zones.

**Methods:**

In coastal and inland regions of Zhejiang Province, China, samples of spot urine, drinking water, and household salt for cooking from both pregnant women and school-age children were determined for iodine concentration between 2017 and 2018. Thyroid-stimulating hormone (TSH) levels from neonates born between 2014 and 2015 were acquired from the Newborns Screening Information System. The iodine status of the vulnerable populations was assessed according to the criteria recommended by the World Health Organization.

**Results:**

The median UIC of pregnant women was significantly lower in the coastal region (113.0 μg/L) than the inland region (134.9 μg/L; *p* < 0.001). The median UICs of pregnant women from these two regions were below the lower optimal iodine cutoff level of 150 μg/L. The percentage of neonates with elevated TSH (> 5 mIU/L) was significantly higher in the coastal region (15.8%) than the inland region (10.5%; *p* < 0.001). The percentage of neonates with elevated TSH from each region decreased within the range of mild iodine deficiency of 3–19.9%. The median UIC of the coastal school-age children was 156.0 μg/L, and the median UIC of inland children was 181.5 μg/L. Both medians fell within the recommended optimal iodine range of 100–299 μg/L. The iodine concentrations in drinking water varied from 1.0 μg/L in the inland region to 2.0 μg/L in the coastal region. The proportion of households that consumed iodized salt was lower in the coastal region (nearly 65%) than the inland region (approximately 95%).

**Conclusions:**

In these two regions with low iodine contents in drinking water, both pregnant women and neonates were iodine-deficient, although school-age children were iodine-sufficient. Urgent efforts are needed to improve the iodine status of pregnant women and neonates.

## Introduction

Iodine is an essential component of thyroid hormones for brain development [1]. Humans need to meet the iodine requirement via their diet since iodine cannot be produced in vivo. Insufficient iodine intake from the diet may induce iodine deficiency disorders (IDD), which have adverse health effects on humans [2]. The most distinct adverse effect of IDD is irreversible mental retardation and low IQ in the early stage of life [3–5], which impair intellectual capacity and school performance. Pregnant women, fetuses, and young children are the most vulnerable populations to IDD [6].

China once had a severe IDD endemic. IDD affected approximately 425 million people in the 1980s, accounting for 40% of the total affected population across the world [7]. China introduced a universal salt iodization (USI) program in 1995. Periodic surveillance of the iodine status of vulnerable populations is essential to refine the USI program to ensure optimal iodine status. At the beginning of the USI program, the population of school children between 8 and 10 years of age was the only focus because school children are easily accessible for epidemiological investigations. Substantial achievement towards the elimination of IDD was made until 2005. The proportion of households consuming iodized salt increased from less than 50% before 1995 [8, 9] to 95% in 2005 [5], and the median urinary iodine concentration (UIC) of school-age children increased from 50 μg/L to higher than 100 μg/L during the same period [8–10].

With the development of the USI program, the iodine status in the pregnant population has been of a main concern in national IDD surveillance since 2011. The national IDD surveillance results from 2014 [11] showed that pregnant women were iodine-deficient in the eastern provinces (i.e., Zhejiang, Fujian, and Guangdong) whereas pregnant women had adequate iodine levels in the western provinces (i.e., Shanxi and Jiangsu). Regional disparities of the iodine status in pregnant women pose a question whether geographic influences in the iodine status of vulnerable populations occur in the current context of the USI program. Moreover, studies on the iodine status in neonates remain sparse.

The aim of this study was to describe the iodine status in pregnant women, neonates and school-age children from a coastal city and an inland city in Zhejiang, and to assess the content of iodine in both household salt for cooking and drinking water from these different areas.

## Materials and methods

In this study, Taizhou and Lishui were selected as the coastal and inland regions, respectively, because data on neonates’ TSH values were available in only these two regions. Data on the TSH concentration in heel-prick blood collected 3 to 7 days after birth between October 2014 and September 2015 were acquired from the National Newborns Screening Information System, which provided the TSH values for more than 98% of live births [12]. Additional information on the newborn’s geographical area, birthdate, and screening dates were extracted. A total of 84,004 newborn TSH concentrations were obtained, with 63,274 from the coastal region and 20,730 from the inland region.

For pregnant women and school-age children, stratified random sampling was performed. The sampling followed the WHO’s and Chinese IDD surveillance guidelines [6, 13], and the details have been provided in our previous studies [14, 15]. Briefly, a total of eighteen counties from these two regions were selected. For each selected county, five towns were randomly chosen. For each selected town, a total of 40 children aged 8 to 10 years old were selected from one public primary school, and 21 pregnant women who routinely visited antenatal care clinics were invited and recruited from March 2017 to December 2018. For each participant, 5 mL morning spot urine and 30 g household salt for cooking samples were obtained. A total of 3884 school-age children participated in this study and submitted samples of urine and household salt for cooking. A total of 1923 pregnant women participated in this study, of whom 107 self-reported a historical diagnosis of thyroid diseases. The final number for analysis was 1797 pregnant women since each of them provided samples of morning spot urine and household salt for cooking.

For each selected town in these two regions, drinking water samples were collected according to the categories of the water supply system. If drinking water was supplied through pipelines, one sample from the source of the water supply was collected. If drinking water was from wells, five drinking water samples were obtained from five wells in different geographical locations (east, west, south, north, and center). If drinking water was supplied via both ways, sampling was performed from both pipeline water and wells. A total of 290 towns were selected, with 162 towns from the inland and 128 towns from the coastal regions. A total of 1155 drinking water samples from the water pipe sources and 201 samples from the wells were obtained to determine the iodine concentration.

UIC was examined using the colorimetric ceric ion arsenious acid method. The iodine concentration in salt was measured using the titrimetric method with sodium thiosulphate. The iodine concentration in drinking water samples was determined via the As3^+^-Ce4^+^ catalytic spectrophotometric method. All of the iodine laboratories participated in internal quality control and external quality assurance programs run by the Chinese Centers for Disease Control and Prevention (CDC). The thyroid volume of children was measured by trained investigators with a 7.5-MHz transducer ultrasound device.

In this study, the criteria for assessing the iodine status recommended by the World Health Organization (WHO)/United Nations Children’s Fund (UNICEF)/International Council for Control of Iodine Deficiency Disorders (ICCIDD) were adopted [6, 16].In detail, the iodine status was assessed based on the median UIC in pregnant women or school-age children and thyroid-stimulating hormone (TSH) concentration in neonates. The iodine status of pregnant women was defined as deficient if the median UIC was below 150 μg/L and sufficient if between 150 μg/L and 249 μg/L. The iodine status of school-age children was classified as deficient if less than 100 μg/L and sufficient if between 100 μg/L and 199 μg/L. Newborns with elevated TSH concentration (above 5 mIU/L) between 3 and 19.9% were mildly iodine-deficient, whereas newborns with elevated TSH of less than 3% were iodine-sufficient. Additionally, school-age children with a goiter prevalence of less than 5% were also considered to be iodine-sufficient.

SPSS software (version 25; Chicago, IL, USA) was utilized for all analyses. Continuous data following a normal distribution were displayed as the mean and standard deviation (SD), e.g., age and gestational weeks. The means between the coastal and inland regions were compared using *t*-tests. Continuous data following a non-normal distribution were expressed as the median and interquartile range (IQR), e.g., UIC, iodine content in drinking water, iodine concentration in household salt for cooking, and TSH level. The medians from two different groups (such as the coastal and inland regions, iodized salt and non-iodized salt) were analyzed using Mann-Whitney tests. For categorical data (the percentage of neonates with elevated TSH levels or goiter incidence), the number and percentage are shown, and Chi-squared tests were used for comparison of the two groups (the coastal and inland regions). A *p-*value of less than 0.05 was determined to be statistically significant.

## Results

The demographic characteristics of the vulnerable populations by region are shown in Table 1. Of the 1797 pregnant women who participated, their ages ranged from 15.8 to 47.0 years, and gestation ranged from 3 to 40 weeks. There were no significant differences in age (*p* = 0.083) or gestation (*p* = 0.295) between the inland and coastal regions. Of the 84,004 neonates, the percentage of girls was significantly higher in the inland region than in the coastal region (*p* = 0.001). No difference in the mean age between the coastal and inland regions was observed (*p* = 0.200). For the 3884 school-age children, no significant differences were observed in gender (*p* = 0.775) or age (*p* = 0.572) between these two regions.

**Table 1:** Demographic characteristics of vulnerable populations by region

Population	Demographic characteristics	Inland	Coast
Pregnant women	N	895	902
	Age, mean ± SD (years)	30.4 ± 5.2	29.9 ± 4.9
	Gestational age, mean ± SD (weeks)	22.3 ± 9.1	21.8 ± 9.3
	Trimester, N (%)		
	1 (≤13 weeks)	184 (20.6)	229 (25.4)
	2 (14–27 weeks)	403 (45.0)	378 (41.9)
	3 (≥28 weeks)	308 (34.4)	295 (32.7)
Neonates	N	20,730	63,270
	Girls, N(%)^*^	9759 (47.1)	29,222 (46.2)
	Age, mean ± SD (days)	3.6 ± 0.8	3.6 ± 0.8
School-age children	N	1948	1936
	Girls, N(%)	995 (51.1)	980 (50.6)
	Age, mean ± SD (years)	8.7 ± 0.8	8.9 ± 0.8

^*^: *p < 0.05*

Table 2 shows the median UICs for the 1797 pregnant women who participated stratified by region and by category of salt consumed. In general, the median UIC of the pregnant women in coastal regions (113.0 μg/L) was significantly lower than those in the inland regions (134.9 μg/L; *p* < 0.001). Both medians were below the WHO/UNICEF/ICCIDD-recommended lower cutoff criteria for optimal iodine status of 150 μg/L [1]. The UICs of 585 (64.9%) pregnant women from the coastal region and 507 (56.6%) pregnant women from the inland remained below 150 μg/L. The median iodine concentration of household salt for cooking from a total of 1797 pregnant women was 23.9 ppm (IQR 23.6–24.0 ppm), with no significant difference between the two regions (*p* = 0.176). In the inland region where 95.3% (853/895) of households consumed iodized salt, no significant difference in the median UIC was observed between those who consumed iodized salt and those who used non-iodized salt (*p* = 0.272). Nevertheless, in the coastal region where 61.9% (558/902) of households used iodized salt, the median UIC for those who consumed iodized salt was significantly higher than that for those who consumed non-iodized salt (*p* < 0.001). For the households that consumed non-iodized salt, the median UIC was significantly higher in the inland region than in the coastal region (*p* < 0.006). No significant difference in the median UIC for the households that consumed iodized salt was defined by region (*p* = 0.272).

**Table 2:** Median UIC for pregnant women by region and category of household salt for cooking

Category of household salt for cooking	Median (IQR), μg/L	
	Inland	Coast
Iodized	135.4 (85.1–205.1)^1,3^	133.0 (78.5–196.2)^2**^
Non-iodized	127.8 (75.2–180.5)^4*^	93.7 (60.2–136.7)
Total	134.9 (85.0–203.0)	113.0 (69.5–180.1)

^1^: the median UIC for the inland pregnant women consuming iodized salt was compared with the median UIC for those inland consuming non-iodized salt at a household level

^2^: the median UIC for the coastal pregnant women consuming iodized salt was compared with the median UIC for those coastal consuming non-iodized salt at a household level

^3^: the median UIC for the inland pregnant women consuming iodized salt was compared with the median UIC for those coastal consuming iodized salt

^4^: the median UIC for the inland pregnant women consuming non-iodized salt was compared with the median UIC for those coastal consuming non-iodized salt

^*^: *p* < 0.05; ^**^: *p* < 0.001

The median TSH concentration in the neonates from the coastal region was 2.49 mIU/L (IQR 1.42–4.03 mIU/L), which was significantly higher than that in neonates from the inland region (2.17 mIU/L, IQR 1.26–3.47 mIU/L; *p* < 0.001). The percentage of TSH above 5 mIU/L was 15.8% in the coastal region and 10.5% in the inland region, respectively, each falling into the range of mild iodine deficiency recommended by the WHO/UNICEF/ICCIDD (3–19.9%) [6].

For the 3884 school-age children who participated, the median UICs distributed by region or by category of household salt consumed are shown in Table 3. The median UIC for inland children (181.5 μg/L) or coastal children (156.0 μg/L) fell into the WHO/UNICEF/ICCIDD-recommended range of optimal iodine levels of 100–299 μg/L [6]. In addition, in the coastal region where 64.3% (1244/1936) of households consumed iodized salt, those consuming iodized salt had a significantly higher median UIC (170.1 μg/L; *p* < 0.001) than those consuming non-iodized salt (132.0 μg/L). In the inland region where 95.1% (1853/1948) of households used iodized salt, however, the median UIC for those who consumed iodized salt (182.8 μg/L) was not significantly different from that for those who used non-iodized salt (158.6 μg/L; *p* = 0.989). For the households who consumed non-iodized salt, the median UIC was significantly higher in the inland school-age children than in the coastal children (*p* = 0.047). For the households who consumed iodized salt, the median UIC in the inland school-aged children was not significantly different from that in the coastal school-aged children (*p* = 0.272). The median iodine concentration of the 3884 samples of household salt for cooking was 23.8 ppm (IQR 23.7–24.0 ppm). No significant difference was observed by region (*p* = 0.633).

**Table 3:** Median UIC for school-age children by region and category of household salt for cooking

Category of household salt for cooking	Median (IQR), μg/L	
	Inland	Coast
Iodized	182.8 (117.2–250.5)^1,3^	170.1 (115.1–235.0)^2**^
Non-iodized	158.6 (115.0–213.6)^4*^	132.0 (83.1–192.1)
Total	181.5 (117.0–249.7)	156.0 (103.0–222.0)

^1^: the median UIC for the inland school-age children consuming iodized salt was compared with the median UIC for those inland consuming non-iodized salt

^2^: the median UIC for the coastal school-age children consuming iodized salt was compared with the median UIC for those coastal consuming non-iodized salt

^3^: the median UIC for the inland school-age children consuming iodized salt was compared with the median UIC for those coastal consuming iodized salt

^4^: the median UIC for the inland school-age children consuming non-iodized salt was compared with the median UIC for those coastal consuming non-iodized salt

^*^: *p* < 0.05; ^**^: *p* < 0.001

Among the 3884 participating school-age children, the thyroid gland volumes of 1708 children were measured. Goiter prevalence was 3.9% (25/637) for inland children and 4.2% (45/1071) for coastal children. Goiter prevalence for each region was lower than the national criteria for IDD elimination of 5.0%. No significant difference in goiter prevalence was observed between the inland and coastal regions (*p* = 0.780).

The median iodine concentration in drinking water at the town level was calculated and is shown in Table 4. In general, the median iodine content in drinking water from each town was low, slightly fluctuating between 1.0 and 1.2 μg/L. According to the national criteria for the classification of regions [17], these two regions are classified as low-iodine areas because the medians fall into the recommended range of less than 10 μg/L.

**Table 4:** Median iodine concentration in drinking water by category of water supply system and region

Category of the water supply system	Inland		Coast	
	N	Median (IQR), μg/L	N	Median (IQR), μg/L
Pipelines	11	1.0 (0.8–1.9)	49	2.3 (1.9–3.2)
Mixture	134	1.0 (0.7–1.2)	77	2.0 (1.3–2.4)
Wells	17	0.5 (0.4–1.0)	2	0.7 (0.7–0.7)
Total	162	1.0 (0.6–1.2)	128	2.0 (1.5–2.7)

## Discussion

To the best of our knowledge, this is the first study to extensively investigate the iodine content in drinking water and household salt for cooking and the iodine status of pregnant women, newborns, and school-age children in Zhejiang Province, China, after the introduction of the new salt iodine concentration of 25 ppm. The results of this current study have showed that pregnant women and neonates remain iodine-deficient, although school-age children are iodine-sufficient in the regions with low iodine contents in drinking water.

The iodine contents in drinking water from both the inland and coastal regions were low, fluctuating from 1.0 μg/L in the inland region to 2.0 μg/L in the coastal region. If an average amount of 1.0–1.5 L water per capita is consumed daily, then the iodine intake from drinking water would be 1.0–3.0 μg per day, which appears negligible when compared with the iodine intake of 90 μg per day for school-age children or 230 μg per day for pregnant women recommended by the National Nutritional Association of China [18]. These results indicate that iodine intake from drinking water was too low to maintain the daily iodine requirements for the inland and coastal populations. Because it is challenging to improve the iodine content in drinking water, we suggest that the salt iodization program should be continuously implemented in these regions because iodized salt is safe and effective for the prevention and control of IDD [19].

In this study, the percentages of households from the coastal region who used iodized salt were lower than those from the inland region (pregnant women: 61.9% in the coastal region vs. 95.3% in the inland region; school-age children: 64.3% in the coastal region vs. 95.1% in the inland region). The geographical variations in the percentage of households using iodized salt may be related to the differences in knowledge and attitude regarding the consumption of iodized salt between coastal and inland inhabitants. Our previous study showed that inhabitants from the coastal areas had a widespread incorrect perception that iodine-rich seafood (e.g., seaweed and marine fish) was more natural and safer than iodized salt when asked why seafood was chosen as the preferred source of iodine [20]. Consequently, costal inhabitants who consume seafood are reluctant to buy iodized salt. Another previous study from our team also showed that consumption of iodized salt represented the majority of dietary source of iodine (approximately 75%), whereas consumption of seaweed or marine fish contributed only approximately 15 and 1% of the dietary sources of iodine, respectively [21, 22]. In addition, the percentage of households that utilize iodized salt in the inland region has reached the national lowest cutoff criteria of IDD elimination of 95%. The percentage of households in the coastal region that utilize iodized salt, however, is now at the lowest level since the adoption of USI, from 92.7% in 2005 [10] to 88.4% in 2014 [11] and below 65% in this current study.

This study also shows that the pregnant population remain iodine-deficient when consuming 25 ppm iodized salt. This finding is in line with our previous IDD surveillance results from pregnant women in Zhejiang Province [14, 15]. Nevertheless, the finding in this study is not consistent with the results from the three other provinces (Shandong, Shanxi and Jiangsu), where pregnant women were iodine-sufficient when they consumed 25 ppm iodized salt [11].. The potential reason for the differences between regions may be related to the geographical varieties in the iodine concentrations of drinking water [23–28]. Gao and his colleagues have shown that the iodine concentrations in drinking water across Shandong Province range from 1.0 to 400.0 μg/L [29]. High iodine contents in drinking water (higher than 100 μg/L) have been found to be scattered across Shanxi and Jiangsu [24–26]. These abovementioned studies have shown that the median UIC in the population is positively correlated with the iodine content in drinking water under the current USI program [29, 30]. The median UIC for pregnant women increased from 197.7 μg/L to 221.9 μg/L and to 452.1 μg/L when the iodine content in drinking water gradually increased from 3.0 μg/L to 57.5 μg/L and to 464.0 μg/L, respectively.

This study shows that inland pregnant women had a median UIC of 134.9 μg/L, and the percentage of inland newborns with elevated TSH was 10.5%. When compared with those above indicators from the inland region, a lower median UIC (113.0 μg/L) was present in the pregnant women from the coastal region, and a higher percentage of coastal newborns with elevated TSH was observed (15.8%). Because an elevated TSH level in neonates may represent inadequate thyroid hormone levels during brain development and have adverse effects on the IQ score [3, 31], more urgent efforts need to be made to improve the iodine status in both pregnant women and neonates.

Our findings show that goiter prevalence for school-age children (4.2% from the coastal region and 3.9% from the inland region) fell within the range of the national criteria of IDD elimination of less than 5%, reflecting iodine sufficiency for school-age children in recent years. In addition, for inland children who consumed either iodized salt or non-iodized salt, the median UIC steadily remained between 100 and 199 μg/L. This finding indicates that school-age children remains iodine-sufficient even when they did not use iodized salt at home, which is in line with the study conducted in Shanghai [32]. It is presumed that the major dietary source of iodine may not be from home-salt-prepared foods, but other commercial foods prepared with iodized salt, e.g. processed foods and packaged foods, since iodization of all food-grade salt is required according to the current well-operated USI program. Considering that most school-age children have lunch offered by the primary school cafeterias on weekdays, we also presume that daily intake of iodine for these children is mainly from iodized salt in the cafeterias.

Iodization of all food-grade salt, which includes household salt for cooking and cooking salt at schools, is recommended by the USI program; however, the recommendation that at least 95% of households using iodized salt identified by this program may not be essential to correct IDD in school-age children whose diets are mainly prepared at home and at school. For pregnant women, improving vigilance is needed to increase the proportion of households that consume iodized salt and improve iodine intake. Further studies are needed to optimize the USI program to ensure optimal iodine status for both pregnant women and neonates.

This study has certain limitations. First, the inland participants without consuming iodized salt at home had a better iodine status than those coastal participants without consuming iodized salt. However, it is not well understood in this study. With the standard of living improved greatly, an increasing amount of the population choose foods outside the home and possibly supplemented iodine through consuming iodized salt from processed foods and packaged foods. Iodine content of salt for these foods has not been clarified and need to be monitored in the future. Second, IDD has adverse health effects on humans during all life stages. More specific populations (e.g., young infants, reproductive women, and lactational women) should be covered in future studies.

## Conclusions

USI remains the primary strategy by which to achieve the goal of IDD elimination in populations residing in regions with low iodine concentrations in drinking water. After the iodine concentration in household salt for cooking was modified to 25 ppm, the salt iodization program boosted iodine intake in school-age children to sufficiency but failed to improve the iodine status of pregnant women and neonates to an optimal iodine level. More urgent efforts should be made to improve iodine intake by pregnant women and neonates because even mild IDD during pregnancy would have adverse effects on fetal brain development.

## Data Availability

The datasets supporting the conclusions of this article are available from the corresponding author on reasonable request.

## Abbreviations

CDC: Center for disease control and prevention
ICCIDD: International council for control of iodine deficiency disorders
IDD: Iodine deficiency disorders
IQR: Interquartile range
SD: Standard deviation
TSH: Thyroid-stimulating hormone
UIC: Urinary iodine concentration
UNICEF: United Nations children’s fund
USI: Universal salt iodization
WHO: World health organization
